# Easter Eggs

**Published:** 1887-04-16

**Authors:** 


### EASTER EGGS.

The festival of the Resurrection probably derives its name from the festival of the goddess Ostara or Eastre, who seems to have been the personification of the morning, or east, and also of the opening year, or spring. In the ancient Church the celebration of Easter lasted eight days, and tradition warrants us in to-day calling the reader's attention to the opportunities Easter offers to all for the display of good works. In the earliest times, at this season, alms were dispensed to the poor and needy, who were even feasted in the churches. Slaves also received their freedom; and as the austerities of Lent were over, and the people gave themselves up to enjoyment, they shared their rejoicings with the poor, and especially with the sick. The most characteristic Easter rite, and the one most widely diffused, was the use of Pasch, that is, Easter Eggs.

The Jews used eggs in the feast of the Passover; and it is asserted that the Persians, when they keep the festival of the solar new year in March, present each other with coloured eggs. Easter eggs were usually stained of various colours with dye woods or herbs. It has been the custom in some parts of Scotland for young people to rise early and search for wild fowls' eggs for breakfast, and it was thought lucky to find them. The Easter egg, from time immemorial, has been presented, or preserved, as a keepsake and omen of good fellowship and good fortune. There can be little doubt that the use of eggs at Easter was originally symbolical of the revivification of nature—the springing forth of life in spring. Christians have regarded this feast of eggs as emblematic of resurrection and of a future life; and it is from this point of view that we wish to regard it to-day. Easter being the time of general rejoicing, of presents, of new departures and new efforts to live a better life, caused the day to be called the Sunday of Joy. Sunday, as the first day of the week, was commended by the Apostles to the members of the Christian Church as a day of almsgiving and helpfulness to those in need. Christian people, who have zealously endeavoured to profit by the season of Lent, by religious observances, by abstinence, by a stricter mode of living and an inward examination of their own methods and modes of conduct, enter upon Easter with a heart-felt feeling of thankfulness and joy. Christmas is often regarded as the season for gifts, but we make bold to think that, if evidence could be obtained, it would be found that Easter is the season of seasons in England for dispensing the largest sums in gifts and donations for charitable purposes. To the zealous Churchman and Nonconformist, and to the thoughtful man or woman, no exhortation will be necessary to ensure that they will make the Sunday of Joy, and the days immediately following it, the occasion for good works and special liberality. So we will content ourselves with reminding them that the Great Physician himself commended the sick and the suffering to the care of His followers, and that the hospitals are greatly in need of funds at the present time.

The force of example is contagious, and imitation is held to be the sincerest form of flattery. May we not, therefore, hope that all who have read to this point, who may not yet have been contributors to the hospitals, will exhibit awakened interest in the thoughts here expressed, and so be led to subscribe something to the hospitals as an experiment in good works? The luxury of giving has been wisely extolled by preachers and speakers for generations, and those who will risk a little money in the experiment, and ascertain the personal benefit derived from an indulgence in this luxury, will not fail to be satisfied that they have selected a good investment for their money. We have often felt that preachers, when appealing to their congregations in their charity sermons, make a mistake when they urge their hearers to loosen their purse-strings, and pour their gold indiscriminately at the feet of charity. Each man and each woman has a distinct personal responsiblity in proportion to the amount of this world's goods with which Providence has blessed them. It is for each individual to decide whether or not they will gather the flowers which the charities that soothe, and heal, and bless, have scattered at men's feet. Such golden opportunities for exhibiting the fruits of a thankful heart do not too often recur, and cannot be too often or too gladly embraced.

The season of Easter, symbolical, as we have seen, of life and revivification of nature, is one such opportunity. The present year is the Jubilee of Queen Victoria, than whom no monarch has set a nobler example to the charitable, or done more to win the gratitude and the love of her people. This must not be forgotten, and cannot fail to stimulate the grateful sentiments of a grateful people to special acts of beneficence at this time. The Laureate has seized this idea in his Ode, and has urged all who are capable of making the Jubilee real and happy to give gold to the hospitals, and to let the maimed in heart rejoice. There is not a London hospital which does not need additional aid, and all who give of their substance to the hospitals will have the satisfaction of feeling that the Easter egg which they thus bestow will be memorable as the commencement of a line of conduct, which the more they pursue it, the more they will find it helpful and satisfying year by year.

